# Key determinants of deposits volume using CAMEL rating system: The case of Saudi banks

**DOI:** 10.1371/journal.pone.0261184

**Published:** 2021-12-15

**Authors:** Dania AL-Najjar, Hamzeh F. Assous

**Affiliations:** Finance Department, School of Business, King Faisal University, Al Hofuf, Saudi Arabia; Szechenyi Istvan University: Szechenyi Istvan Egyetem, HUNGARY

## Abstract

CAMEL is considered one of the well-known banking rating systems used to build a proper bank ranking. In our paper, we investigate the CAMEL rating for Saudi banks, which is considered the second largest banking sector in GCC. The Saudi banking sector consists of 11 banks and is the leading sector in the Saudi stock index (TASI). In this research, we aim to determine the ranking of Saudi banks according to CAMEL composite and CAMEL overall ratings and explore the effects of these ratings on banks’ total deposits for the period from 2014 to 2018. The methodology involves four phases. In the first phase, we calculate the key financial ratios of CAMEL’s composites for each bank. In the second phase, we rank the banks from 1 to 11 to each one of CAMEL’s composites for each bank per year. In the third phase, we rank Saudi banks according to CAMEL composite and CAMEL overall. Finally, in the fourth phase, we run a regression model using CAMEL financial ratios rank as independent variable and banks’ total deposits as a dependent variable. Using the stepwise regression method, the results indicated that the best regression model has an adjusted R^2^ of 73.4% and a standard error of around 0.58. The results further indicated that capital measured by CAR, management as an efficiency ratio, earning with ROE proxy, and liquidity as loans to deposits have positive effects on banks’ total deposits. Meanwhile, earnings as net interest income to net revenue and liquidity calculated by CASA have a negative effect on banks’ total deposits. Finally, asset quality ratios and the rest of the ratios have no significant effect on banks’ total deposits.

## Introduction

Banks are the key financial performers in economies and the mirror of all other sectors. The banking system plays a vital role in the economy as an important channel through which cumulative investments increase. The genuine development of the banking sector’s actions promotes economic activities and its growth by encouraging savings and mobilizing public savings. Thus, when the banking sector performs well, the whole economy will succeed.

Banks underpin the modern economy and play a central role in the transmission of monetary policy, which in turn enhances stability and economic growth. The importance of banks comes from their role as financial institutions that accept deposits from the public to use them in many banking products, mainly by offering loans to their customers to earn interest. Deposits and loans are crucial figures on the banks’ balance sheets. Deposits are relatively the cheapest source of funds for banks and loans are the main use of funds in banks. However, deposits cannot be increased without the strong financial position of banks. Extra deposits could enhance the bank’s trust while the increase in loans should only be attached to highly rated clients.

Rating systems are very important for predicting the potential bankruptcy of different parties. Banks apply one of these systems to assess the creditworthiness of their clients scientifically and accurately and to predict the possibility of bankruptcy of those clients in advance [1–3].

Central banks apply the rating system because they are responsible for managing the country’s financial system in general and regulating banks. Hence, central banks’ rating for banks specifies the level of direct supervision it requires and enhances the depositors’ trust in banks. The central bank’s regulations are crucial for the proper functioning of economies and societies and for preventing banks from engaging in risky activities or repeating similar mistakes that could threaten the banking sector and the entire economy. Regulations should also improve financial stability and encourage clean competition among banks [4–6].

The concept of examining the banks’ processes and operations was first applied through Uniform Financial Institutions Rating System for banks in the USA. Regulatory bodies and central banks worldwide adopted CAMEL as a supervisory rating system to evaluate and differentiate between strong and distressed banks and to specify the required level of supervision required for each bank.

The CAMEL rating system is the abbreviation of the five assessment composites, namely, Capital, Asset Quality, Management Quality, Earnings Quality, and Liquidity. These ratings are very important for depositors because they can enhance the trust in banks and protect depositors’ wealth.

Many researchers have applied the CAMEL rating system and CAMEL composites in their research to assess and rank public, private, conventional, and Islamic banks [7–14].

In our study, we aim to use the ranks of CAMEL composites and the overall rank of Saudi banks to determine the drivers of bank deposits. Saudi banking sector consists of 11 banks, seven of which are conventional and the rest are Islamic. The Saudi baking system is considered the second largest banking sector in GCC with a total asset of 29% of the region’s total banking assets. The Saudi banking sector index is the leading sector index in TASI.

To the best of our knowledge, this paper is the first to study the effects of CAMEL ranking on Saudi banks’ total deposits. The main contributions of this paper are (I) it ranks each bank according to the CAMEL composites ratios from 1 to 11 for the period from 2014–2018 (II) it ranks each bank according to CAMEL composites and overall ranking on average basis for the period from 2014–2018, (III) it specifies which of the CAMEL composites has the strongest effect on Saudi banks’ total deposits, and (IV) it highlights the most important theoretical background and literature review of CAMEL ratings and banking sector.

This study is structured as follows. Section 2 describes the literature review, Section 3 discusses the methodology, Section 4 shows the main results of our analysis, and Section 5 provides the conclusions.

## Literature review

Banks are the backbone of any economy and are completely interlinked with the financial systems that existed in the economies [15]. Although banks that perform in the same country have the same environment, significant variations in their performance can be observed as found in [16].

Many researchers have attempted to rank banks and assess them using different models. [17] assessed the credit risk of public and private banks by applying multiple models (i.e., Altman Z-score, Springate, and Grover and Zmijewski models). [18] studied banking Z-score and found that distressed stocks outperform non-distressed stocks during market growths.

### CAMEL overall and banks’ performance

CAMEL’s overall rating for banks has a scale from rate 1 to rate 5. Rate 1 indicates that banks have a strong performance and excellent risk management practices, while rate 5 indicates the lowest rating with the worst performance and lack of risk management practices.

The CAMEL model has been used by many researchers to rank public and private banks. [8, 10] investigated the financial performance of public and private banks to assign ranks based on the five CAMEL composites and overall ranks. According to [11], private banks were superior in three CAMEL composites, namely, asset quality, earnings quality, and management efficiency, while public banks performed better under the liquidity composite. However, [15] found that public banks have focused on increasing their capital composite to reach a suitable level of capital but need to determine more creative ideas to employ their funds and to maximize their profits. However, [13] found that private banks perform better than public banks only in management efficiency and earnings quality, while in other CAMEL composites, both types of banks have the same level.

Other researchers have investigated the difference between conventional and Islamic banks using the CAMEL model. [7, 12, 14] found that conventional banks outperformed Islamic banks according to the CAMEL model.

Finally, the CAMEL model is a well-known model used by many researchers to rank the financial performance of banks and is applied by many central banks worldwide to assess banks’ position. [9] found that the CAMEL model can be used to build an early warning system for banks’ failure.

### CAMEL composites and bank performance

CAMEL composites rating gives the bank per each composite a rate from one to five, as for Capital, rate 1 means strong capital level while 5 means a critical deficient level of capital.

Researchers found that banks obtained different ranks when they are rated using one of CAMEL composites than when the CAMEL overall model is used [19–23].

#### Capital adequacy “C” and bank performance

Capital adequacy indicates the level of banks’ compliance with regulations of the minimum capital reserve amount. The capital structure concentration for any bank is highly important, as shown in [24], whose findings revealed that the number of large and institutional shareholders of banks has a positive effect only on profitability, not on risk.

Several scholars have studied bank Capital ratios (i.e. Capital Adequacy Ratio) and found a significant and positive relationship/effect of capital on profitability proxies and major key financial performance indicators [25–29]. [30] proved the existence of a positive relationship between CAR and return of deposits money banks. CAR has a strong effect on the change in loans and a positive effect on lending [31, 32]. [33] investigated the relation between CAR ratios and efficiency, and revealed that the efficiency ratio has a positive effect on CAR in Islamic banks and a negative effect on CAR in conventional banks.

[34] showed that the performance of banks is significantly influenced by banks’ decisions related to capital, cost control, business diversification, asset quality, and liquidity.

#### Asset and loan quality and bank performance

Many researchers investigated the importance of “A” Asset / Loan Quality in banks because loans are the core portion of banks’ assets. Asset quality is an important figure in banks as the value of assets can decrease rapidly if they are at high risk. Asset/Loan quality discusses the way that banks manage their assets and loans to maximize the income of these assets and minimize non-performing loans and non-performing assets. Researchers found that poor asset quality and high non-performing loans affect profitability ratios and other KPIs negatively [12, 35–37]. Moreover, [38] investigated the effects of non-performing assets on the financial stability and profits of public banks. The study concluded that non-performing loans affect the financial position of the banking and non-banking financial companies.

[39] investigated product development projects’ financing choices under traditional initial coin offerings and traditional bank loans in the blockchain era and concluded that financing models have an important effect on optimal pricing, initial profits, and quality decisions.

To enhance the asset quality of banks, [40] found that quarterly financial reports of banks improve loan and asset quality. [41] found that banks with lower asset quality will benefit more from income diversifications (i.e. non-interest income) and will result in increasing the profit. [42] concluded that banks should increase non-performing loans provisions and banks must build sound and proactive units to efficiently manage non-performing loans to become performing loans. [43] found that non-performing loans increase the chance of bank distress and thus, building enough provisions can be a good action to mitigate this distress probability. Finally, [44] findings showed that conventional banks have high-quality assets and stability compared to Islamic banks.

#### Management quality and banks’ efficiency

Management quality measures the quality of a bank’s business strategy financial performance and internal controls. Several scholars examined the importance of “M” Management Quality in banks. [45] showed that management quality and ROE have a strong influence on the probability of banking crises. [46] revealed that conventional banks have better management and asset quality compared to Islamic banks who have better CAR and liquidity ratios. [47] found that the big five Chinese banks suffer from low average cost efficiency while [48] found that a significant heterogeneity in Chinese commercial banking efficiency for overall efficiency, productivity, and profitability efficiency.

[49] determined the cost-efficiency ratio and non-performing loans are significantly negatively related to financial performance. [50] indicated that bank size and the number of bank branches were important drivers of bank efficiency and further found that capital, asset, and earnings of banks were essential factors for technical efficiency and pure technical efficiency of the banks.

Other scholars have explored banks’ management quality through the implementation of best corporate governance and social responsibility practices [24, 51–53]. [51] applied different corporate governance practices in banks (i.e., female independent directors, CEO duality, and CEO shareholding) and found that bank financial performance was positively affected by these practices. [53] revealed that social responsibility and human resource management have a significant positive effect on bank reputation and a significant negative influence on turnover intention.

#### Earning quality

The importance of “E” Earnings quality is to evaluate the banks’ long-term viability because they need an appropriate return to be able to grow their operations and maintain their competitiveness.

Earning quality has been explored by many researchers. [54] found that bank size and age, intellectual capital performance, and barriers to entry have a significant effect on earnings quality. [55] revealed that earnings management practices are highly influenced by audit committee techniques, and concluded that earning management is lower in Islamic banks compared to conventional banks.

However, [56] found that banks with high earnings management practices will encourage audit committees to increase their voluntary disclosure. [57] revealed that higher earnings management practices of banks were caused by lower foreign ownership and higher ownership concentrations of these banks. Finally, [58] showed that banks’ earnings and insolvency risk are extremely affected by sustainable banks practices while market power was not one of the profitability drivers in sustainable banks.

#### Liquidity

The last composite is “L” liquidity. The liquidity of banks is a vital concept as the lack of liquid capital can lead to a bank run. According to [59], a moderate increase in banks’ liquidity help in enhancing their efficiency, while too much liquidity could increase the inefficiency level of the bank. [60] showed that liquidity and solvency risk factors positively affect cost efficiency measures. [61] found that Islamic banks’ liquidity is positively affected by CAR ratios while negatively affected by credit risk and profitability ratios. Nevertheless, [62] analyzed the economic structure of ethical and conventional banking, and they found that ethical banking is growing more with greater liquidity and solvency levels but almost the same profitability.

[63] revealed that bank connections within a network are important to understand how banks set their liquidity ratios. Other scholars concentrate on the type of deposits to enhance liquidity. [64] showed that if a bank’s deposits are less than the bank’s advances, the bank will have a problem with liquidity. However, [65] found that the credit deposit ratio (segregated into banks-high credit deposit ratio and banks-low credit deposit ratio) has a significant effect on the profitability of the banks.

### Banks deposits and CAMEL composites

Deposit mobilization is the first step in the financial intermediation process. Banks cannot function without deposits because these deposits are cheap and reliable sources of funds for development in countries. Banks should finance more of their loans from deposits so that the bank will not face liquidity squeezes and enhance banking system stability.

CAMEL rating is important in enhancing the ability of banks in attracting new deposits. Many researchers have explored the effects of macroeconomic and bank-specific factors to determine the drivers of total deposits. [66] examined the determinants of banks deposits for the period from 2008 to 2017 using random effects. The findings showed that the profitability, bank’s size, profitability, and liquidity are the most significant determinants of bank deposits.

[67] defined the determinants of Moroccan bank deposits for the period 2003–2014 using panel data regression. Results showed bank risk, interest rate, and bank size as significant variables of deposits. [68] study the determinants of deposit mobilization using panel least regression and fixed effects for a sample of 112 banks. The results revealed that loan to asset ratio, liquidity ratio, and bank size are the most significant drivers of deposit mobilizations.

[69] analyzed the commercial banks’ deposits and found that it is positively related to bank profitability. [70] found that interest rate and the real rate of return affected saving accounts in Islamic banks. [71] found that bank-specific factors (i.e liquidity, bank risk, and loan exposure) influence deposits.

Hence, we aim to address the following questions:

1. What are the financial ratios of CAMEL composites?
2. What are the ranks of conventional and Islamic Saudi banks using CAMEL overall ranking and CAMEL composites?
3. Can CAMEL overall ranking and CAMEL composites determine the drivers of Saudi banks’ deposits?

## Methodology

To answer our research questions, we have first to specify the CAMEL ranking for Saudi banks. Hence, the central bank of Saudi Arabia imposed clear and sharp regulations regarding the soundness and strength of Saudi banks to specify the level of supervision and follow-up needed in each bank. All banks have high ranking and excellent financial ratios. We will simulate the CAMEL model ranking by analyzing specific financial ratios for each composite. Next, the Saudi banks will be ranked upon these financial ratios, which will then be rated according to each CAMEL composite. Finally, Saudi banks will be ranked using CAMEL overall rank.

The methodology will be implemented as follows. The first stage is to prepare the data by calculating the main financial ratios of CAMEL’s composites for each bank for the period from 2014–2018. Then, to provide ranking from 1 to 11 for the average of each financial ratio for each bank within the period under study. The second stage involves the analysis part that includes ranking the Saudi banks according to the CAMEL composite and CAMEL overall ranking. Then, the regression model is run using the CAMEL financial ratios ranking as independent variables and the banks’ total deposits as a dependent variable.

### Data collection

First, we started by calculating the financial ratios of CAMEL model composites, namely, Capital, Assets Quality, Management Quality, Earnings Quality, and Liquidity. There are eleven Saudi banks (seven conventional and four Islamic banks). The CAMEL composites of these banks were translated into 13 indicators and covered the period from 2014–2018.

To explore the effects of the CAMEL model on banks’ total deposits, we started by calculating the financial ratios of each bank for the period from 2014–2018.

Capital ratios include total capital adequacy ratios CAR and CAR tier 1. Asset quality ratios include loan losses to total loans (LL/TL) and loan losses to total equity (LL/TE). For Management ratios, we implemented net profit per employee, efficiency ratio, and earnings growth. Moving to earnings ratios are calculated by ROA, ROE, net interest income to total assets (NII/ TA), and net interest income to net revenue (NII/NR). Finally, we used loans to deposits (LTD) and current and saving counts to total deposits (CASA) as proxies of liquidity.

## Ranks based on CAMEL financial ratios

We will use the calculated ratios for the period from 2014–2018 to calculate the arithmetic average per ratio for each bank. We then give ranks for banks from 1 to 11 according to each average ratio, in which rank 1 reflects the bank that has the best ratio and rank 11 indicates the bank that has the lowest ratio, this methodology is used by Singhal, 2020.

Table 1 shows the averages of ranks per each ratio for the period under consideration. According to Basel III Accords, capital is measured by capital adequacy ratios (total and tier 1), in which CAR ratios are calculated by dividing a bank’s capital on its risk-weighted assets. A bank’s capital consists of tiers I, II, and III, in which Tier 1 consists of shareholders’ equity and retained earnings. The denominator of CAR ratios is the risk-weighted assets containing three types of risk: operational, credit, and market risk.

**Table 1:** Ranks of Saudi banks using CAMEL composites (C, A, M).

	CAR Total	CAR Tier 1	LL/TL	LL/TE	Net Profit Per Employee	Efficiency Ratio	Earnings Growth
Alinma Bank	1	1	1	1	4	9	1
Al Rajhi Bank	2	3	10	10	9	3	5
Samba Financial Group	3	2	4	2	1	1	10
Saudi British Bank	5	4	9	7	2	2	6
Riyad Bank	8	5	2	3	8	6	9
National Commercial Bank	6	6	8	8	6	7	8
Banque Saudi Fransi	9	7	7	6	3	4	4
Saudi Investment Bank	7	9	5	4	5	8	11
Bank Aljazira	4	8	3	5	10	11	2
Arab National Bank	11	10	6	9	7	5	7
Bank Albilad	10	11	11	11	11	10	3

CAR (total, tier 1) ratios are imposed by central banks because of the recommendations of Basel Accords, in which total CAR and tier 1 should not be less than 10.5% and 6%, respectively. CAR ratios are very essential to regulatory bodies because they ensure the ability of banks’ capital to absorb a reasonable amount of loss and protect the banks from taking an extra risk or becoming insolvent. These ratios will protect depositors and enhance the soundness and stability of the financial sector not only in the country but also worldwide. According to our analysis in Table 1, Alinma Bank had the highest CAR ratios while Arab National Bank and Bank Albilad had the lowest CAR and CAR tier 1, respectively.

Banks’ asset includes cash, government securities, interest-earning loans, and investments. Asset quality of banks and Loan quality are two expressions with essentially the same meaning. The quality of Assets in banks means the quality of loans, which can be reflected in enhancing the soundness and profitability for the bank. Loans are classified into performing loans (PL) and non-performing loans (NPL). NPL refers to loans in which the borrower did not pay the scheduled payments for 90 days. In our study, we used loan losses ratios that indicate the loss that banks have when loans are not paid back. According to Asset quality proxies, as shown in Table 1, Alinma Bank had the best Assets ratios, Loan losses to total loans (LL/TL), and loan losses to total equity (LL/TE) as compared to Bank Albilad, which had the least ranking.

In Management ratios, we focused on the ratios that measure the management’s ability in directing the main activities of the bank and the funds. Management ratios include efficiency ratio that indicates the ability of the banks to utilize their funds efficiently. The managing capabilities of maximizing and increasing the profits and earnings of banks are measured by net profit per employee, efficiency ratio, and earnings growth.

Our analysis shows that Samba Financial Group had the highest net profit per employee and the best efficiency ratio. Bank Aljazeera and Bank Albilad had the lowest efficiency ratio and Net profit per employee, respectively. Finally, Alinma Bank had the highest earning growth compared to Saudi Investment Bank with the lowest ratio.

Table 2 shows that Earnings are the most important KPIs in any institute, while ROA and ROE are considered vital ratios that reflect profitability. In addition to the previously mentioned ratios, banks have special earnings ratios of net interest income to total assets (NII/TA) and net interest income to net revenue (NII/NR). Net interest income is considered the most important figure in banks’ income statements and their main source of income. Accordingly, by using the proxies of ROA, ROE, and NII/TA, AlRajhi Bank has the highest ratios while Aljazeera Bank, Alinma Bank, and Saudi Investment Bank have the lowest ratios of ROA, ROE, and NII/TA, respectively. According to NII/ NR, Alinma Bank had the highest and Samba Financial Group had the lowest figure.

**Table 2:** Ranks of Saudi banks using CAMEL composites (E, L).

	ROA	ROE	NII/TA	NII/NR	LTD	CASA
Alinma Bank	8	11	3	1	1	7
Al Rajhi Bank	1	1	1	4	7	1
Samba Financial Group	3	7	9	11	11	3
Saudi British Bank	2	3	4	6	6	5
Riyad Bank	6	9	5	7	3	8
National Commercial Bank	4	2	2	2	10	2
Banque Saudi Fransi	5	6	10	5	8	6
Saudi Investment Bank	10	10	11	3	2	11
Bank Aljazira	11	8	8	9	9	10
Arab National Bank	7	5	7	8	5	9
Bank Albilad	9	4	6	10	4	4

Liquidity refers to the ability of banks to pay back their current liabilities from their current assets. Bank’s Liquidity ratios include Loans to deposits (LTD) and Current and saving counts to total deposits (CASA). Table 2 shows that Alinma Bank had the best LTD ratio versus Samba Financial Group, which had the lowest LTD. CASA, AlRajhi Bank had the best ratio compared to Saudi Investment Bank, which had the lowest ratio.

## Analysis and results

In this section, we rank the banks according to each CAMEL composite and overall ranking, then run a regression model to find the effect of the CAMEL ranking of Saudi banks on total deposits.

## Ranks based on CAMEL composites and overall rank

To rank the Saudi banks according to CAMEL composites and CAMEL overall ranking, we computed the average of the ranks of each financial ratio for each composite of CAMEL composites. As for capital, we took the average of the “CAR and CAR tier 1” ranking for each bank then ranked the bank according to capital composite as shown in Table 3.

**Table 3:** Overall ranking of Saudi banks using CAMEL.

Bank	C	A	M	E	L	CAMEL
Alinma Bank	1.00	1.00	4.67	5.75	4.00	3.28
Al Rajhi Bank	2.50	10.00	5.67	1.75	4.00	4.78
Samba Financial Group	2.50	3.00	4.00	7.50	7.00	4.80
Saudi British Bank	4.50	8.00	3.33	3.75	5.50	5.02
Riyad Bank	6.50	2.50	7.67	6.75	5.50	5.78
National Commercial Bank	6.00	8.00	7.00	2.50	6.00	5.90
Banque Saudi Fransi	8.00	6.50	3.67	6.50	7.00	6.33
Saudi Investment Bank	8.00	4.50	8.00	8.50	6.50	7.10
Bank Aljazira	6.00	4.00	7.67	9.00	9.50	7.23
Arab National Bank	10.50	7.50	6.33	6.75	7.00	7.62
Bank Albilad	10.50	11.00	8.00	7.25	4.00	8.15

Table 3 shows that in the CAMEL composite ranking, Alinma Bank had the highest Capital and Assets ratios, while Saudi British Bank had the highest Management quality ratios. In the Earnings ratio, AlRajhi Bank had the highest average earnings ratios. Alinma Bank, AlRajhi Bank, and Bank Albilad had the highest Liquidity ratios.

Finally, to rank the banks according to CAMEL’s overall ranking, we calculated the average of the five CAMEL composites. The calculations indicated that Alinam Bank had the highest CAMEL overall ranking followed by Al Rajhi Bank, while Bank Albilad had the lowest CAMEL overall ranking.

## Regression analysis

We run the regression model- stepwise method as shown in Tables 4 and 5 to study the effects of CAMEL ranking of Saudi banks on total deposits. The best model is Model 6, which is significant with an adjusted R^2^ of 73.4% and a standard error of around 0.58. Table 4 also shows that the autocorrelation test -Durbin Watson is equal to 1.122.

**Table 4:** Model summary.

Model	R	R Squared	Adjusted R Squared	Std. Error of the Estimate	Durbin Watson
1	.692^a^	.479	.470	.81918	
2	.785^b^	.616	.601	.71032	
3	.821^c^	.674	.654	.66134	
4	.844^d^	.712	.689	.62725	
5	.858^e^	.736	.709	.60652	
6	.874^f^	.764	.734	.58013	1.122

^a^Predictors: (Constant), Efficiency Ratio.

^b^Predictors: (Constant), Efficiency Ratio, CASA.

^c^Predictors: (Constant), Efficiency Ratio, CASA, NII/NR.

^d^Predictors: (Constant), Efficiency Ratio, CASA, NII/NR, LTD.

^e^Predictors: (Constant), Efficiency Ratio, CASA, NII/NR, LTD, CAR.

^f^Predictors: (Constant), Efficiency Ratio, CASA, NII/NR, LTD, CAR, ROE.

^g^Dependent Variable: Deposits.

**Table 5:** ANOVA^a^ test of the linear regression model.

Model		Sum of Squares	df	Mean Square	F	Sig.
1	Regression	32.759	1	32.759	48.817	.000^b^
	Residual	35.566	53	.671		
	Total	68.326	54			
2	Regression	42.089	2	21.044	41.708	.000^c^
	Residual	26.237	52	.505		
	Total	68.326	54			
3	Regression	46.020	3	15.340	35.073	.000^d^
	Residual	22.306	51	.437		
	Total	68.326	54			
4	Regression	48.653	4	12.163	30.915	.000^e^
	Residual	19.672	50	.393		
	Total	68.326	54			
5	Regression	50.300	5	10.060	27.347	.000^f^
	Residual	18.025	49	.368		
	Total	68.326	54			
6	Regression	52.171	6	8.695	25.836	.000^g^
	Residual	16.154	48	.337		
	Total	68.326	54			

a. Dependent Variable: Deposits.

The regression analysis was run using a stepwise method as shown in Table 6. The table shows that the multicollinearity problem does not exist because each variable has a VIF figure less than 10. The results also showed that there are six models, and that Model 6 is the best model with the highest adjusted R^2^ and lowest standard error.

**Table 6:** Coefficients of the linear regression model.

Model		Unstandardized Coefficients		Standardized Coefficients	T	Sig.	VIF
		B	Std. Error	Beta			
1	(Constant)	10.017	.237		42.283	.000	
	Efficiency Ratio	.244	.035	.692	6.987	.000	1.000
2	(Constant)	11.335	.369		30.715	.000	
	Efficiency Ratio	.173	.035	.490	5.005	.000	1.299
	CASA	-.148	.035	-.421	-4.300	.000	1.299
3	(Constant)	11.656	.360		32.393	.000	
	Efficiency Ratio	.195	.033	.553	5.908	.000	1.367
	CASA	-.137	.032	-.389	-4.236	.000	1.317
	NII/NR	-.087	.029	-.246	-2.998	.004	1.052
4	(Constant)	11.094	.404		27.426	.000	
	Efficiency Ratio	.163	.034	.462	4.840	.000	1.581
	CASA	-.095	.035	-.270	-2.743	.008	1.684
	NII/NR	-.099	.028	-.280	-3.543	.001	1.081
	LTD	.096	.037	.271	2.587	.013	1.907
5	(Constant)	10.821	.412		26.279	.000	
	Efficiency Ratio	.183	.034	.518	5.396	.000	1.712
	CASA	-.110	.034	-.313	-3.213	.002	1.759
	NII/NR	-.107	.027	-.303	-3.926	.000	1.103
	LTD	.084	.036	.239	2.333	.024	1.950
	CAR	.060	.028	.170	2.116	.039	1.204
6	(Constant)	10.383	.436		23.837	.000	
	Efficiency Ratio	.199	.033	.565	6.012	.000	1.792
	CASA	-.172	.042	-.487	-4.096	.000	2.872
	NII/NR	-.109	.026	-.310	-4.196	.000	1.105
	LTD	.084	.035	.237	2.418	.019	1.950
	CAR	.093	.030	.263	3.040	.004	1.515
	ROA	.089	.038	.252	2.358	.023	2.317

a. Dependent Variable: Deposits

Table 6 further shows that the Capital composite (measured by CAR) has a positive effect on total deposits. High CAR indicates a high level of efficiency and stability because it lowers the risk of banks’ insolvency and banks can meet their financial obligations. Accordingly, high CAR ratios increase the trust of depositors to deposit more in this bank. Our results of the positive significant effect of Capital ratios on total deposits are not consistent with [66]. who found a negative insignificant effect on total deposits.

Table 6 also shows the Management composite has a positive effect on management (measured by efficiency ratio) on total deposits. Efficiency is one of the most important KPIs for banks because it reflects the bank’s ability to utilize funds and deposits effectively. An efficient utilization will result in enhancing the profitability of banks and maximizing the stockholders’ wealth.

Earnings are defined as the reflection of good management and an efficient way of managing their funds. Table 6 shows the positive effect of earnings (measured by ROE) on total deposits and this result is consistent with Haron et al. (2006). Our analysis showed a negative effect of earnings (measured by NII/NR) on total deposits, which is consistent with [66] who found a negative effect on the profitability of total deposits.

Liquidity indicates the ability of banks to pay their short-term liabilities using short-term assets, and our findings revealed that a significant effect of liquidity on total deposits, which agrees with the findings of [71]. The results showed a positive effect of liquidity (measured by LTD) on total deposits. However, the results indicated a negative effect of liquidity (measured by CASA) on total deposits which is consistent with [66] who found a negative effect on total deposits. Nevertheless, our findings indicated that the asset quality composite has no significant effect on total deposits.

## Conclusion

The goal of our study is to investigate the effects of the CAMEL ranking on total deposits using Saudi banks’ financial ratios for the period from 2014 to 2018. We used 13 ratios to reflect the CAMEL ranking, which includes the following. Total capital adequacy ratios proxies, including CAR and CAR tier 1. Assets quality ratios, which include LL/TL and LL/TE. For Management ratios, we implemented net profit per employee, efficiency ratio, and earnings growth. Moving to earnings ratios are calculated by ROA, ROE, NII/TA, NII/NR. Finally, we used LTD and NON-IID/ TD as proxies of liquidity.

According to the data analysis, the capital ratios (CAR and CAR tier 1) indicated that Alinma Bank had the highest CAR ratios while Arab National Bank and Bank Albilad had the lowest ratios. Alinma Bank has the best Assets ratios. For loan losses to total loans (LL/TL) and loan losses to total equity (LL/TE), Bank Albilad had the least ranking.

Samba Financial Group has the highest net profit per employee and the best efficiency ratio, while Bank Aljazeera and Bank Albilad have the lowest efficiency ratio and net profit for employees, respectively. Finally, Alinma Bank had the highest earnings growth, while Saudi Investment Bank had the lowest-earning growth.

Furthermore, using the proxies of ROA, ROE, and NII/TA, Al Rajhi Bank was found to have the highest ratios while Aljazeera Bank, Alinma Bank, and Saudi Investment Bank had the lowest ratios. Alinma Bank had the highest NII/NR compared to Samba Financial Group, which has the lowest ratio.

ALinma Bank has the best LTD ratio versus the Samba Financial Group, which had the lowest ratio. For CASA, Al Rajhi Bank had the best ratio as compared to Saudi Investment Bank, which had the lowest ratio.

Finally, CAMEL composite ranks on average found that Alinma Bank had the highest Capital and Assets ratios, while Saudi British Bank had the highest Management quality ratios. In the earnings ratio, AlRajhi Bank had the highest average earnings ratio. Alinma Bank, AlRajhi Bank, and Bank Albilad had the highest Liquidity ratios. Alinam Bank had the highest CAMEL overall rank while Bank Albilad had the lowest CAMEL overall rank.

A regression model using the stepwise method was run to specify the significant CAMEL composites on Saudi banks’ total deposits. The best model with the highest adjusted R-squared and lowest standard error had a positive effect on capital (measured by CAR) and management (measured by efficiency ratio) on the bank’s total deposits. However, the mixed result of earnings was determined because of the positive effects of earnings (measured by ROE) and negative effects of earnings (measured by NII/NR) on total deposits.

Based on liquidity, our findings revealed mixed results because a positive effect was observed (measured by LTD) on total deposits and a negative effect (measured by CASA) on total deposits. Nevertheless, our findings indicated a non-significant effect of asset quality composite on total deposits.

## Limitations and future studies

Our study has certain limitations. First, we depend mainly on CAMEL composites quantitative data to rank banks with no focus on qualitative aspects of the data. Thus, future studies may conduct the same analysis on other GCC banks to strengthen and support our findings. Researchers may also apply the CAMELS model after adding the sensitivity composite for the same sample to improve the results. Finally, further studies can apply the same CAMEL ratings on different GCC banks. We may also apply the effect of CAMEL rating on different banks’ KPIs using other models (i.e., the GARCH model and the artificial neural network).

## Practical implications

Bank depositors may benefit from the conclusion of this study in enhancing the trust in the Saudi banking sector. Investors can also benefit from investing in the stocks of these strong banks to gain extra returns.

Moreover, policymakers of the Saudi central bank and regulatory bodies can take advantage of this study and the ranks that were given to banks in building banks’ early warning systems. This system will ease the supervision procedures of different regulatory bodies on all banks, which will be reflected in enhancing the soundness and strength of the banking sector and the stability of the economy.

## Supporting information

S1 File (XLSX)
